# Better safe than sorry—Whole-genome sequencing indicates that missense variants are significant in susceptibility to COVID-19

**DOI:** 10.1371/journal.pone.0279356

**Published:** 2023-01-20

**Authors:** Dawid Słomian, Joanna Szyda, Paula Dobosz, Joanna Stojak, Anna Michalska-Foryszewska, Mateusz Sypniewski, Jakub Liu, Krzysztof Kotlarz, Tomasz Suchocki, Magdalena Mroczek, Maria Stępień, Paweł Sztromwasser, Zbigniew J. Król

**Affiliations:** 1 National Research Institute of Animal Production, Balice, Poland; 2 Department of Genetics, Biostatistics Group, Wrocław University of Environmental and Life Sciences, Wrocław, Poland; 3 Central Clinical Hospital of Ministry of the Interior and Administration in Warsaw, Warsaw, Poland; 4 Department of Haematology, Transplantation and Internal Medicine, University Clinical Centre of the Medical University of Warsaw, Warsaw, Poland; 5 Department of Experimental Embryology, Institute of Genetics and Animal Biotechnology, Polish Academy of Sciences, Magdalenka, Poland; 6 Department of Genetics and Animal Breedings, Poznan University of Life Sciences, Poznan, Poland; 7 Center for Cardiovascular Genetics & Gene Diagnostics, Foundation for People with Rare Diseases, Schlieren-Zurich, Switzerland; 8 Department of Infectious Diseases, Doctoral School, Medical University of Lublin, Lublin, Poland; 9 MNM Diagnostics, Poznań, Poland; Nigerian Institute of Medical Research, NIGERIA

## Abstract

Undoubtedly, genetic factors play an important role in susceptibility and resistance to COVID-19. In this study, we conducted the GWAS analysis. Out of 15,489,173 SNPs, we identified 18,191 significant SNPs for severe and 11,799 SNPs for resistant phenotype, showing that a great number of loci were significant in different COVID-19 representations. The majority of variants were synonymous (60.56% for severe, 58.46% for resistant phenotype) or located in introns (55.77% for severe, 59.83% for resistant phenotype). We identified the most significant SNPs for a severe outcome (in *AJAP1* intron) and for COVID resistance (in *FIG4* intron*)*. We found no missense variants with a potential causal function on resistance to COVID-19; however, two missense variants were determined as significant a severe phenotype (in *PM20D1* and *LRP4* exons). None of the aforementioned SNPs and missense variants found in this study have been previously associated with COVID-19.

## Introduction

The established risk factors of coronavirus disease 2019 (COVID-19) are advanced age, male sex, and comorbidities [1–3], but they do not fully explain the wide spectrum of disease manifestations [4]. Several studies investigated other factors associated with different COVID-19 outcomes that were either environmental, population-based, or genetic. As demonstrated by numerous association studies, the genetics of the host can influence the difference in response to infection and therefore, different clinical presentations of COVID-19. To date, several genetic variants that are associated with susceptibility and resistance to COVID-19 were identified. They are associated with ancestry, virus entry, receptors, and immunological processes.

Risk variants are located in multiple genes. Ellinghaus et al. [5] described two regions associated with risk variants, one on chromosome 3 that harbours multiple genes (*SLC6A20*, *LZFTL1*, *CCR9*, *CXCR6*, *XCR1*, and *FYCO1*) and a region on chromosome 9 that defines the ABO blood groups. Zeberg and Paabo [6] showed that a genomic segment associated with severe COVID-19 was inherited from the Neanderthals, and was carried by around 50% of people in South Asia and around 16% of people in Europe. Other risk variants were reported on chromosomes 6, 12, 19, and 21 [7]. The review of variations across mitochondrial DNA (mtDNA), conducted by Wu et al. [8] points to differences in *ND1*, *ND2*, *ND3* genes and in the D-loop region as related to an increased risk of severe COVID-19. Moreover, it was demonstrated that several gene variants related to cardiovascular and pulmonary diseases were correlated with the severe outcome of COVID-19 [9].

Regions associated with COVID-19 resistance have also been reported, mainly as population-specific. Zeberg and Paabo [10] identified a haplotype on chromosome 12, inherited from the Neanderthals, associated with an approximately 22% reduction in relative risk of severe COVID-19. It is present in all regions of the world except Africa with approximately 25–30% frequency in Eurasian populations and lower frequencies in the Americas. This protective haplotype contains parts or all *OAS1*, *OAS2*, and *OAS3* genes that encode enzymes induced by interferons during the infection with RNA viruses. Interestingly, Papadopoulos et al. [11] reported that the prevalence of haemoglobin E heterozygote, which is widespread in south-eastern Asia and associated with thalassemia syndrome, was positively correlated with immunity to COVID-19. The review of variations across mitochondrial DNA (mtDNA) showed differences in the *ND2* gene, in the D-loop region and in the *cytochrome c oxidase I* gene that was related to a reduced risk of severe COVID-19 [8]. Moreover, the GWAS analysis identified the Allograft Rejection metabolic pathway as associated with resistance to COVID-19 infection [12].

Some risk variants have been identified in genes involved in the immunological processes. Zhang et al. [13] identified ten variants in loci involved in the TLR3- and IRF7-dependent induction and amplification of type I Interferons that increase susceptibility to severe COVID-19 pneumonia. Also, the deleterious variants of the TLR7 contributed to higher susceptibility to COVID-19 in males [14]. Multiple genetic variants accounting for various presentations of COVID-19 were also found in human leukocyte antigens (HLA), proteins encoded by the major histocompatibility complex (MHC) [15]. For instance, the HLA-B*46:*01* variants were demonstrated to influence the susceptibility to severe COVID-19 [16], while the HLA-B*15:*03* variant was indicated as a protective one [16].

The analyses of the *ACE2* gene, which is located in the X chromosome and characterised by a high rate of polymorphism in its coding regions, indicated that different variants contribute differently to COVID-19 infection [17, 18]. The protective *ACE2* variants (*K31R*, *N33I*, *H34R*, *E35K*, *E37K*, *D38V*, *Y50F*, *N51S*, *M62V*, *K68E*, *F72V*, *Y83H*, *G326E*, *G352V*, *D355N*, *Q388L*, and *D509Y*) showed decreased binding to the COVID-19 spike protein as compared to the risk variant (*T921*) [19]. It was demonstrated that TMPRSS2, together with cellular receptor ACE2 and exopeptidase DPP4 are membrane-bound proteins entangled in COVID-19 infection and virus entry into the host cell, and consequently, contribute to the severity of the disease [20–22]. The same association was described for different coding variants in furin *PCSK3*, an enzyme that promotes the proteolytic maturation of proproteins [23].

All aforementioned studies would not be possible without genome-wide association studies (GWAS), widely used for various COVID-19 phenotypes [24, 25]. However, most of the studies are based on a single-marker strategy, where a single-SNP effect is incorporated into the model. Such a method requires the use of the Bonferroni correction, which in this case is a very conservative approach. Especially when using SNPs from whole-genome sequencing (WGS) with the number of genetic markers being very large, the assumptions underlying the Bonferroni correction are violated, since it assumes complete independence between particular tests, which is not the case in WGS-based SNPs due to very high Linkage Disequilibrium (LD) typically existing between closely linked variants. Moreover, when a phenotype is determined by several genes, a single-SNP model leads to a representation of the genetic background only by one variant. This means that effects of other SNPs which potentially influence genetic variation either contribute to the polygenic part or to the residual part of the model—where they are confounded with non-genetic variance components. As a result, GWAS models often lead to poor reproducibility of significant genes reports [26] and the fact that genes with moderate effects are missed due to an elevated type II error caused by conservative multiple testing correction. Since resistance to COVID-19 infection and the risk of a severe infection emerge as oligogenic or even polygenic phenotypes, single-SNP models provide an oversimplified description of their genetic background. An alternative strategy is to use a multi-SNP model, fitting all SNPs simultaneously. The major advantage of the simultaneous inclusion of all SNPs is accounting for the correlation between SNPs, which is due to the LD, introduced into the model by the SNP genotype design matrix. Additionally, using LD in the model may increase the power of detecting correlated causal SNPs [27]. Also, the power of detection of the association between phenotype and genotype in a multi-SNP model is higher than in the single-SNP, where the power is undermined by modest SNP effect sizes, unobserved causal SNPs and a correlation among adjacent SNPs [28]. The next advantage of the multi-SNP model is that the unexplained portion of the genetic variance is lower than in the case of the single-SNP model.

Therefore, the main aim of our study was to identify genetic variants (SNPs) marking genes responsible for the increased susceptibility or resistance to COVID-19 infections focusing not only on high-risk variants but also on SNPs with moderate effects, reflecting the possibly oligogenic mode of inheritance of the infection phenotypes.

## Materials & methods

### Sample collection

Blood samples were collected from 1235 individuals across Poland between April 2020 and April 2021. However, for this analysis, a subset of 1076 samples from unrelated individuals was used. Only individuals without diagnosed severe health disorders (till the moment of sample collection), such as cancer, were qualified for this study. The NEWS (National Early Warning Score) scale has been used for the assessment of all hospitalised patients [29]. Within this cohort, the group with a severe outcome of COVID–19 infection (N = 235) was composed of patients with severe, life–threatening cases of COVID–19 infection including respiratory insufficiency, requiring intensive medical care and artificial ventilation, NEWS>5 and the group resistant to infection (N = 306) was composed of volunteers that did not contract the diseases nor develop any symptoms despite being highly exposed to COVID–19 –this group had multiple antibody blood–based tests conducted to confirm they had no antibodies anti–SARS–CoV–2. Detailed information about the cohort, including demographic and clinical features, can be found in Kaja et al. [30].

### Ethical statement

All participants, or guardians/parents of the participants under 18, provided written informed consent before the collection of blood samples and filling in the clinical data form, which included a questionnaire about the country of origin and chronic diseases. Collected consent forms are stored in the Central Clinical Hospital of the Ministry of Interior and Administration in Warsaw, as instructed by the General Data Protection Regulation (GDPR) act. The ethical approval for the study was obtained from the Ethics Committee of the Central Clinical Hospital of the Ministry of Interior and Administration in Warsaw (decision nr: 41/2020 from 03.04.2020 and 125/2020 from 1.07.2020). The study complies with the 1964 Helsinki declaration and its later amendments and adhered to the highest data security standards of ISO 27001 and the General Data Protection Regulation (GDPR) act.

### Total quality management

The project was carried out under the Total Quality Management (TQM) methodology, which ensures the quality of results and analyses the risk and possible difficulties. TQM requires defining all critical points of the procedures: reference ranges for collected biological material, its preparation, isolation, DNA concentration and quality, genomic sequencing, and quality control of the data. The legal and ethical transparency of the entire project was ensured, including confidentiality, integrity, and impartiality of the data.

### Whole genome sequencing

The whole genomes of 1076 unrelated participants were sequenced in this study. 4ml of K-EDTA peripheral blood from participants were collected according to a standardised Quality Management System protocol. Genomic DNA was isolated from the peripheral blood leukocytes using a QIAamp DNA Blood Mini Kit, Blood/Cell DNA Mini Kit (Syngen) and Xpure Blood Kit (A&A Biotechnology) according to the manufacturer’s protocols. The concentration and purity of isolated DNA were measured using the NanoDropTM spectrophotometer, and the quality of the DNA was evaluated using gel electrophoresis. The sequencing library was prepared by Macrogen Europe (Amsterdam, the Netherlands) using TruSeq DNA PCR-free kit (Illumina Inc., San Diego, California, United States) and 550 bp inserts. The quality of DNA libraries was measured using 2100 Bioanalyzer, Agilent Technologies. The Whole Genome Sequencing (WGS) was performed on the Illumina NovaSeq 6000 platform using 150 bp paired-end reads, yielding a mean depth of coverage of 35.26X in the cohort.

### Pre-processing of whole-genome sequence data

The quality of sequenced reads was assessed using FastQC v0.11.7 (see Web Resources). Thereafter reads were mapped to the GRCh38 human reference genome using Speedseq framework v.0.1.2 [31], encompassing alignment with BWA-MEM 0.7.10 [32], SAMBLASTER v0.1.22 [33] for duplicate removal, and Sambamba v0.5.9 [34] for sorting and indexing. SNPs were identified with DeepVariant v0.8.0 [35] and genotyped with GLnexus v1.2.6-0-g4d057dc [36]. The raw SNP set was edited by removing non bi-allelic SNPs, SNPs with a call rate less than 95% in the analysed sample, SNPs with P-values for the Hardy-Weinberg Equilibrium test below 0.0001, and SNPs with the Minor Allele Frequency under 0.001. SNP pre-processing was done using PLINK 1.9 [37].

### Phenotype encoding

The cohort analyzed in this study was divided into multiple categories: control, resistant, benign, mild, and severe. In the analysis of the resistance to COVID-19 infection the resistant group was coded as “1”, the control group was removed from the analysis, and all the other groups were coded as “0”. In the analysis of the susceptibility to severe COVID-19 infection the severe group was coded as “1”, the control group was removed from the analysis, and all the other groups were coded as “0”.

### Genome-wide association study and SNP functional annotation

To include the information on the covariance between SNPs, which is mainly due to the linkage disequilibrium, a mixed linear model simultaneously fitting additive effects of all SNPs was used:

y=Xβ+Zg+ε

where ***y*** represents a vector of binarised disease status, ***β*** is a vector of fixed effects comprising: a general mean, age at data sampling [years] and gender with a design matrix ***X***, ***g*** is a vector of random additive SNP effects with the pre-imposed normal distribution given by ***g***~*N*(0, ***G***) and the corresponding design matrix ***Z*** containing SNP genotype codes parameterised as 0, 1, or 2 for a homozygous, heterozygous and an alternative homozygous SNP genotype, respectively, ***ε*** represents the residual effect with a pre-imposed normal distribution given by ***ε***~*N*(0, ***R***). The model covariance structure was thus defined by the covariance matrix of SNP effects, $G=I\sigma_{g}^{2}$ and the covariance matrix of the residual effects expressed as $R=I\sigma_{\varepsilon }^{2}$, which resulted in the covariance of ***y*** given by ***ZGZ***^***t***^+***R***. In our study, both variance components were assumed as known and set to: $\sigma_{g}^{2}=0.3\sigma_{y}^{2}$ and $\sigma_{\varepsilon }^{2}=0.7\sigma_{y}^{2}$. The model was fitted using the MIXBLUP software [38].

For testing the hypotheses (*H*_0_: *g*_*i*_ = 0 vs. *H*_1_: *g*_*i*_≠0) the Wald test was used: $W=\frac{{\hat{g}}_{i}}{\sigma_{g}}$. Under *H*_0_ this statistic follows the standard normal distribution. The multiple testing correction was carried out via the Bonferroni approach [39]. Testing was implemented into a custom-written R script. Significant SNPs were functionally annotated with the Variant Effect Predictor software [40] using the sequence ontology classification [41]. Furthermore, the enrichment analysis of significant SNPs was performed by applying the Enrichr online tool [42, 43].

## Results

### Significant SNP-sets

**Fig 1:**
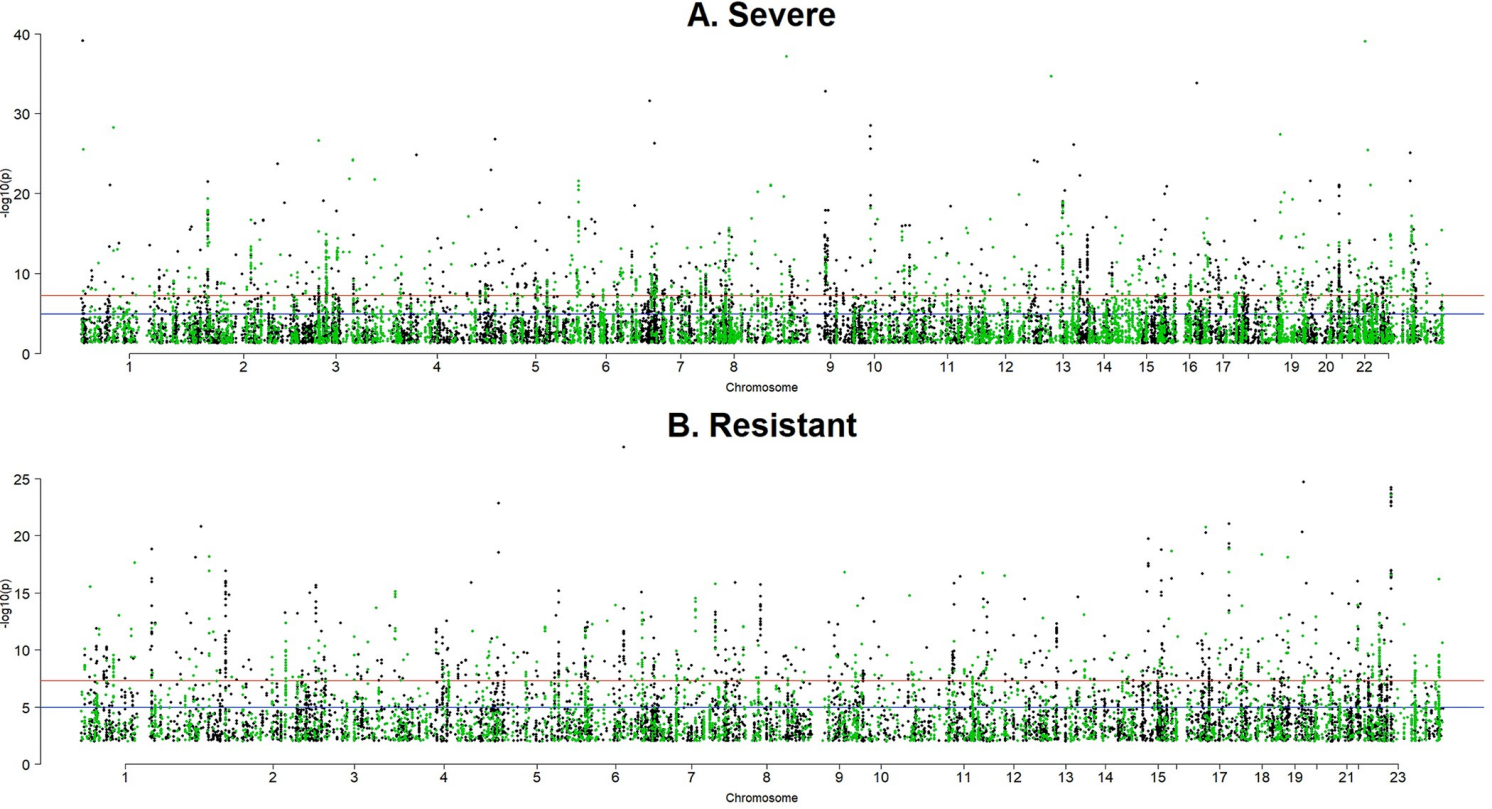
Genome–wide SNP significance for severe (A) and resistant (B) phenotypes with green dots representing SNPs located in exons.

**Fig 2:**
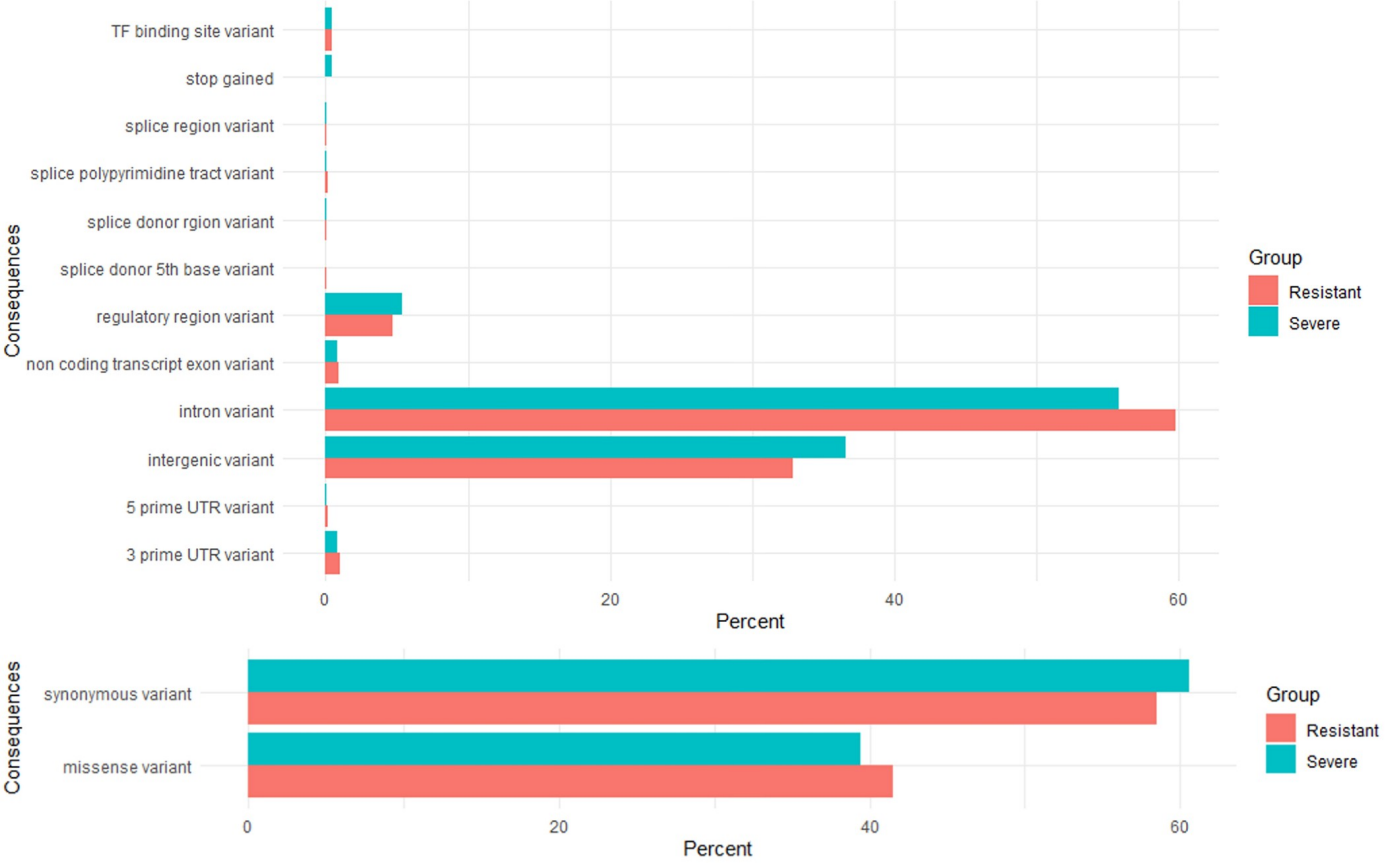
Distribution of significant SNPs for severe (upper panel) and resistant (lower panel) phenotypes, including their genomic annotation (left) and type of mutation for SNPs in coding regions (right).

The original number of the called SNPs amounted to 43,469,928, while, after filtering, 15,489,173 SNPs remained for GWAS. The GWAS analysis demonstrated a large number of significant SNPs and associated genes (Fig 1). In the case of patients that suffered from a severe course of COVID-19, 18,191 SNPs were identified as significant, while in the case of resistant patients, 11,799 SNPs were significant. The majority of significant SNPs were located in introns (55.77% for severe phenotype and 59.83% for resistant phenotype), followed by intergenic regions (36.55% for severe phenotype and 32.92% for resistant phenotype) and regulatory regions (5.41% for severe phenotype and 4.69% for resistant phenotype). The occurrence of SNPs in coding regions was below 1%. Among them, the majority of variants were synonymous (60.56% for severe phenotype and 58.46% for resistant phenotype) (Fig 2).

Missense and synonymous SNPs were annotated to genes and subjected to the enrichment analysis, which however did not reveal any significantly overrepresented functional features. The lists of genes harbouring the significant SNPs for severe and resistant phenotypes were summarised in the S1 and S2 Tables, respectively.

### COVID-19 related significant SNPs

**Fig 3:**
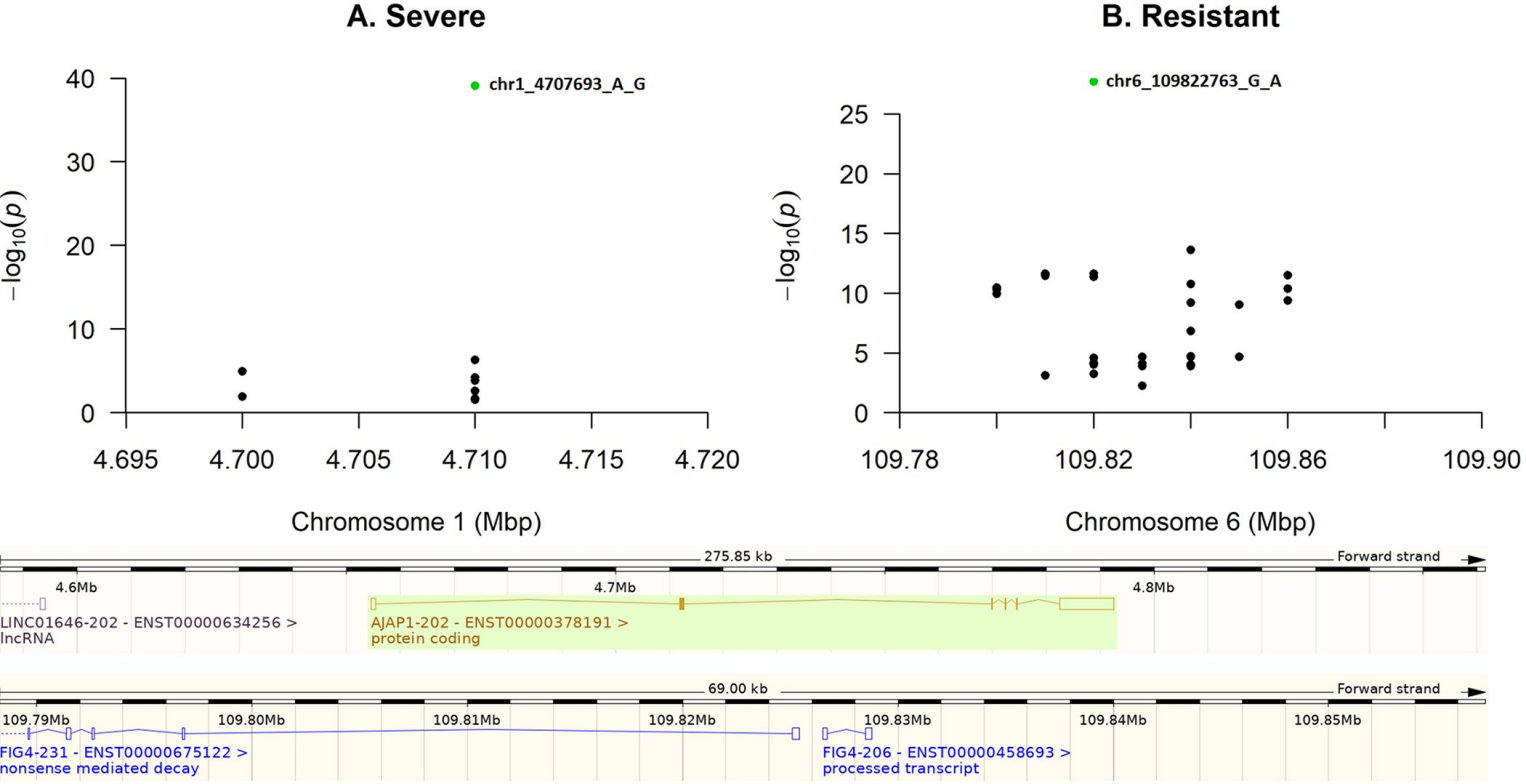
Genomic regions that correspond to the location of the two most significant, intronic SNPs identified for each phenotype, with green dots representing the most significant SNP in severe (A) and resistant (B) phenotypes.

**Fig 4:**
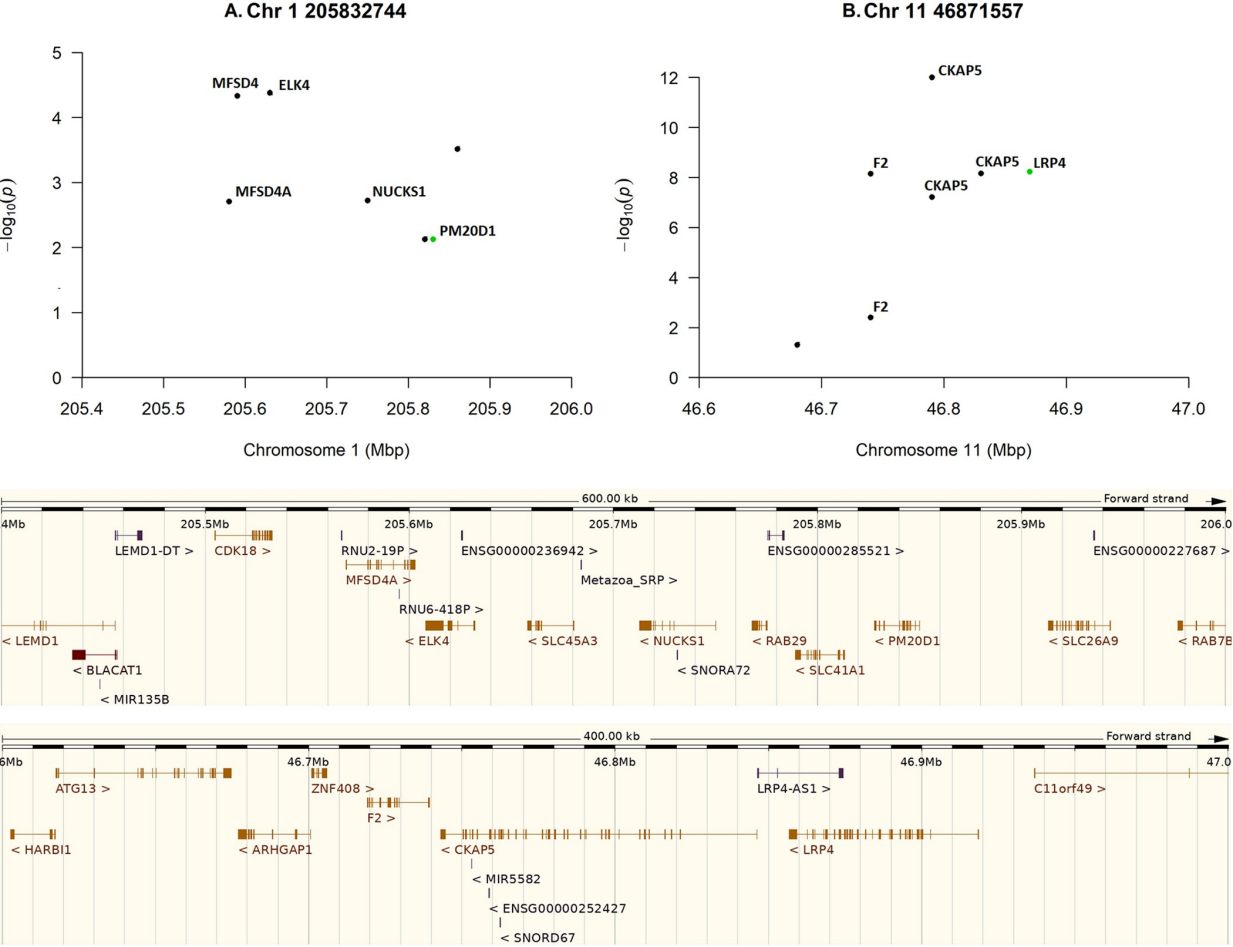
Genomic regions that correspond to the location of two missense SNPs with a potential causal function on a severe outcome of COVID–19, with green dots representing the missense SNPs in severe (A) and resistant (B) phenotypes.

The most significant SNP (rs2101196) for severe outcome was located on chromosome 1, in the intron of adherens junctions-associated protein 1 gene (*AJAP1*) while the SNP (rs7772946) that was the most significant for resistance towards COVID-19 was located on chromosome 6, in the intron of FIG4 Phosphoinositide 5-Phosphatase (*FIG4*). Altogether, the *AJAP1* gene was marked by nine significant, intronic SNPs and *FIG4* by as many as 41 SNPs located in the intron (Fig 3). However, while considering potential causal mutations, we would like to raise attention to two missense variants that pointed at genes with functions that may potentially influence the risk of a severe infection outcome: (i) a variant located in an exon of the peptidase M20 domain containing 1 gene (*PM20D1*) and (ii) a variant located in an exon of the low-density lipoprotein receptor-related protein 4 gene (*LRP4*) (Fig 4, Table 1).

**Table 1:** SNPs with a potential causal function on a severe outcome of COVID–19 infection.

Chromosome	Position / ID	SNP	Gene ID / Transcript ID	Gene name	COVID-19 related phenotypes	P-value	SIFT score
1	205832744 / rs1361754	A/G	ENSG00000162877 / ENST00000367136.5	Peptidase M20 domain containing 1 *PM20D1*	QT interval	0.0075	0.69
11	46871557 / rs2306029	T/C	ENSG00000134569 / ENST00000378623.6	Low-density lipoprotein receptor-related protein 4 *LRP4*	D-dimer level Congenital myasthenic syndrome	6·10^−9^	0.37

## Discussion

COVID-19 infections pose a serious global health concern hence it is crucial to identify biomarkers for the susceptibility and resistance against this disease that could help in a rapid risk assessment and reliable decisions on patients’ treatment and potential hospitalisation. Moreover, COVID-19 manifests a broad spectrum of clinical signs ranging from an asymptomatic to a severe course [44], but we can profit from multiple GWAS results that help researchers determine the impact of genes on mechanisms underlying susceptibility and severity of the COVID-19 infection [45]. D’Antonio et al. conducted GWAS studies and identified four loci with suggestive associations with COVID-19 susceptibility and 19 for COVID-19 disease severity [46]. The GenOMICC (Genetics of Mortality in Critical Care) study has shown some genetic variants significantly predisposing to critical disease, including variants within genes involved in interferon signalling (*IL10RB*, *PLSCR1*), leukocyte differentiation (*BCL11A*), blood type antigen secretor status (*FUT2*), expression of a membrane flippase (*ATP11A*), and mucin expression (*MUC1*) [47]. Another study reported 13 genome-wide significant loci associated with COVID-19 infection and severity (rs2271616, rs10490770, rs11919389, rs1886814, rs72711165, rs912805253, rs10774671, rs1819040, rs77534576, rs2109069, rs74956615, rs4801778, rs13050728). Some of these variants overlap with previously reported associations [48]. Pairo-Castineira et al. identified the following genetic variants associated with a critical outcome of COVID-19: on chromosome 12q24.13 (rs10735079), on chromosome 19p13.2 (rs74956615), on chromosome 19p13.3 (rs2109069), and on chromosome 21q22.1 (rs2236757) [7]. Yet another study indicated 40 genes associated with viral susceptibility, and 21 of them were connected to severe manifestations of COVID-19, including TLR pathways, C-lectin pathways, and inflammasome activation [49].

**Fig 5:**
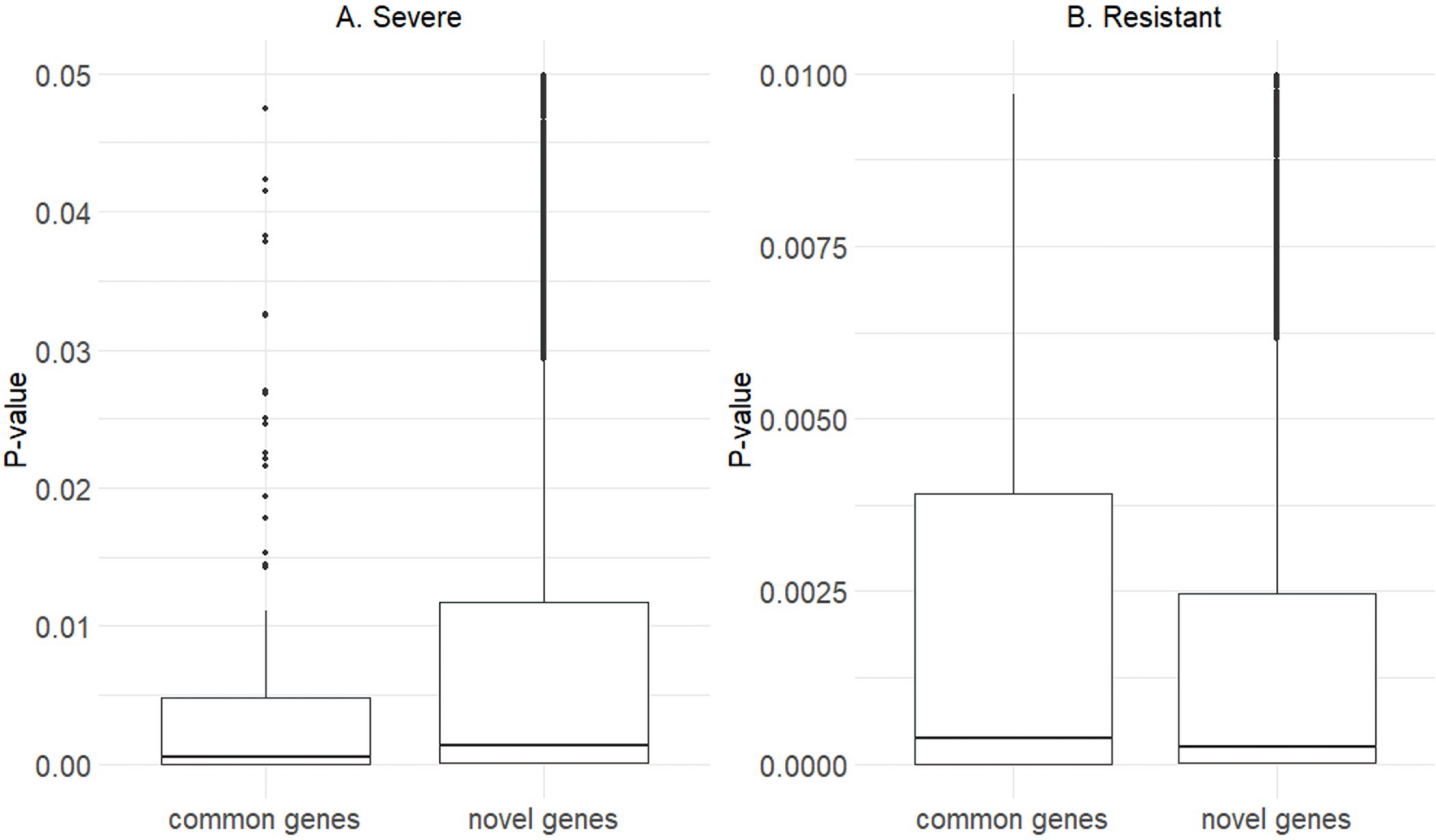
P–values corresponding to groups of SNPs located in genes reported as COVID–19–related in the literature and SNPs located in genes reported as novel by our study.

In our study, we incorporated all SNPs identified using WGS, together in one statistical model with a normal distribution pre-imposed on their effects. By a simultaneous inclusion of all SNPs into the model we accounted for the intercorrelation between them, which was due to LD, which technically was inserted into the model by the SNP genotype design matrix. Therefore, we aimed to select the set of variants that significantly influence the variation in genomes of people who suffered from severe COVID-19 infection and those who never got infected despite exposure (i.e. resistant), focusing on potential causal mutations represented by SNPs located within coding or regulatory regions with high, but also moderate effects. Since the effects of causal variants on phenotypes underlying a complex mode of inheritance are typically moderate to small and many variants are in LD, usage of the whole-genome sequence data and statistical analysis that fits variants simultaneously provides a more realistic statistical handling of the phenotype [50]. As demonstrated in Fig 5, especially for the severe phenotype, as compared to conventional, single-SNP GWAS, the multi-SNP model also identifies SNPs with lower P values, corresponding to lower effects on the response to infection. In general, our analysis identified the number of SNPs significant for severe and resistant phenotypes. The patterns in distribution and type of mutations for these two phenotypes were very similar (Fig 1). The majority of genetic variants were located in introns (over 50%), followed by intergenic regions (over 30%) and a small proportion of variants in regulatory regions (4.5–5%). Interestingly, although the majority of variants were synonymous, there was a considerable number of missense variants, both for severe and resistant phenotypes (39.44% and 41.54%, respectively).

The most significant SNP (rs2101196) for severe outcome was identified on chromosome 1, in the intron of *AJAP1* (Fig 3). AJAP1 is a membrane protein that blocks epithelial–to–mesenchymal transition through interaction with *β*–catenin and inhibiting its nuclear translocation. Overexpression of *AJAP1* may reduce metastasis of hepatocellular carcinoma [51]. In the case of the resistance towards COVID-19, the most significant SNP (rs7772946) was located on chromosome 6, in the intron of *FIG4* (Fig 3). Variants in the *FIG4* gene lead to obstruction of endocytic trafficking, causing the accumulation of enlarged vesicles in murine peripheral neurons and fibroblasts. Therefore, some variants in *FIG4* are related to neurological disorders, such as Charcot-Marie-Tooth disease and Yunis-Varón syndrome [52, 53]. However, none of the above associations seems to be important in the case of COVID-19 related phenotypes.

However, by estimating the effects of all SNPs simultaneously, we mainly focussed on identifying SNPs with potential causal effects, located in coding regions, that may often be missed due to their lower effect on phenotypes as compared to non-coding SNPs being in high LD with such causal mutations. Such SNPs located in coding regions may not list as the top-significant variants because their consequence on phenotypes suppresses their genotypic variability. This typically results in a low frequency of the detrimental allele and hence of the corresponding genotypes in the population. In our sample, no significant missense variants were identified for the resistance to COVID-19, however, two missense variants that pointed at genes with functions that may potentially influence the risk of a severe infection outcome were determined (Table 1). One variant was located in the exon of the peptidase M20 domain containing 1 gene (*PM20D1*) and this particular SNP was associated with the QT interval [54]. QT interval captures the time it takes for the cardiac ventricles to depolarize and repolarize. It was shown that azithromycin and hydroxychloroquine, drugs used in the treatment of COVID-19 could prolong the QT interval on the electrocardiogram, increasing the risk of tachycardia and sudden cardiac death [55]. Other diseases associated with the increased frequency of the *PM20D1* variant are diabetes and obesity [56], and neurodegenerative diseases, such as Parkinson’s Disease [57] and Alzheimer’s Disease [58]. Interestingly two variants in the *PM20D1* have been associated with severe COVID-19 courses requiring ventilatory support and have been located in an epigenome–wide association study [59]. The second missense variant was located in the exon of the low-density lipoprotein receptor-related protein 4 gene (*LRP4*) and two phenotypes, potentially related to COVID-19, were determined for it. This particular SNP was associated with D-dimer levels [60]. COVID-19 is a condition associated with elevated D-dimer. Several studies confirmed this association and showed that D-dimer is elevated in patients with severe COVID-19. The highest D-dimer level was observed in most critically ill patients and those who did not survive [4, 61–63]. This suggests that D-dimer could be a predictor of serious illness and death due to COVID-19. Moreover, this missense variant was also related to postsynaptic congenital myasthenic syndrome [64]. Patients affected by neuromuscular disorders are at higher risk for severe COVID-19 due to respiratory and swallowing muscle weakness [65].

None of identified significant SNPs (in the intron of *AJAP1* and the intron of *FIG4*) and none of the missense variants with a potential causal function on a severe outcome of COVID-19 (in the exon of *PM20D1* and the exon of *LRP4*), found in our study, have been previously associated with COVID-19. It is not surprising, having in mind a generally low overlap between COVID-19 related GWAS. Still, among 1820 genes for severe and 904 genes for resistance marked by SNPs significant in our study, sequentially 20 and 33 have been previously reported, but none have been associated with COVID-19. At this point, we can only hypothesise that the above-mentioned gene discoveries were possible due to the application of the multi-SNP approach, but more dedicated methodological research is needed to (dis)prove the hypothesis.

## Conclusions

Although there are several already established risk factors of COVID-19, such as advanced age, being a male, or coexisting comorbidities, they do not fully explain the wide spectrum of disease outcomes. Undoubtedly, genetic factors also play an important role. Our study presented a solution for searching genes responsible for the increased susceptibility or resistance to COVID-19 infections considering not only high-risk variants but also SNPs with moderate effects, reflecting the possibly oligogenic mode of inheritance of the infection phenotypes. So far, several genetic variants related to ancestry, virus entry, receptors, and immunological processes have been associated with susceptibility and resistance to COVID-19.

In this study, the most significant, albeit non-causal, SNPs pointed at the *AJAP1* gene for a severe outcome (blocking epithelial–to–mesenchymal transition) and the *FIG4* gene for resistance (accumulation of enlarged vesicles in murine peripheral neurons and fibroblasts), while potentially causal, missense variants pointed at two genes with functions that are promising to influence the risk of a severe outcome, namely: missense change in *LRP4* gene could be associated with D-dimer levels and postsynaptic congenital myasthenic syndrome, while missense change in *PM20D1* might be associated with the QT interval.

It’s already been over two years with COVID-19, accumulating data, both clinical and genomic, but we are only at the very beginning of our understanding of the genetic mechanisms underlying the infection. Having huge databases created during the pandemic, now the time has come for more in-depth research to be performed to understand various outcomes of COVID-19 infection and to understand their mode of inheritance, that in view of the relatively low overlap in literature reports published so far, is expected to be of a multi/oligo -genic rather than monogenic.

One of the most limiting factors in applying population-based GWAS to large genomes is the sample size. From the statistical perspective, insufficient sample size leads to elevated type I and type II errors. For genomes composed of millions of base pairs, a large sample size is limited by sequencing costs. Two approaches to enlarge sample sizes emerge: (i) to use a cohort sequenced with high coverage for imputation of variants in a larger cohort that is only genotyped by a microarray [66], (ii) using a cohort sequenced at a moderate coverage without a need of imputation. Both approaches are associated with limitations. Although very broadly used, the former approach does not allow for accurate detection of rare, patient-specific mutations [67], which is not reflected by overall imputation accuracies. The latter approach, which was adopted in our study, is associated with the risk of non-detecting an existing variant due to missing coverage of some nucleotides [68]. Since the available material achieved a moderate genome averaged coverage of over 35X and it is currently the largest sequenced cohort representing the Polish population, the imputation was not considered in our study.

Moreover, in humans, the sample size is also limited by the number of available probands, especially for rare diseases or for disorders that are difficult to quantify–that in the case of our study was the assessment of COVID-19 severity, that could only be conducted in combination to hospital examination and conducting the follow of the environmental (i.e. mainly family) history of each patient.

## Supporting information

S1 Table The lists of genes harbouring the significant SNPs for a severe outcome of COVID-19. (XLSX)

S2 Table The lists of genes harbouring the significant SNPs for resistance to COVID-19. (XLSX)
